# Internal and External Microbial Community of the *Thitarodes* Moth, the Host of *Ophiocordyceps sinensis*

**DOI:** 10.3390/microorganisms7110517

**Published:** 2019-10-31

**Authors:** Yi Liang, Yuehui Hong, Zhanhua Mai, Qijiong Zhu, Lianxian Guo

**Affiliations:** 1 Dongguan Key Laboratory of Environmental Medicine, School of Public Health, Guangdong Medical University, Dongguan 523808, China; 2 Department of Basic Medicine, Guangdong Jiangmen Chinese Medical College, Jiangmen 529000, China

**Keywords:** Chinese cordyceps, *Ophiocordyceps sinensis*, *Thitarodes*, eggs, bacterial community, fungal community

## Abstract

*Ophiocordyceps sinensis* is a widely known medicinal entomogenous fungus, which parasitizes the soil-borne larva of *Thitarodes* (Hepialidae, Lepidoptera) distributed in the Qinghai–Tibetan Plateau and its adjacent areas. Previous research has involved artificial cultivation of Chinese cordyceps (the fungus-caterpillar complex), but it is difficult to achieve large-scale cultivation because the coupling relation between the crucial microbes and their hosts is not quite clear. To clarify the influence of the internal microbial community on the occurrence of Chinese cordyceps, in this study, the unfertilized eggs of *Thitarodes* of different sampling sites were chosen to analyze the bacterial and fungal communities via 16S rRNA and ITS sequencing for the first time. The results showed that for bacteria, 348 genera (dominant genera include *Wolbachia*, *Spiroplasma*, *Carnobacterium*, *Sphingobium*, and *Acinetobacter*) belonging to 26 phyla (dominant phyla include Proteobacteria, Firmicutes, Tenericutes, Actinobacteria, Acidobacteria, and Bacteroidetes), 58 classes, 84 orders, and 120 families were identified from 1294 operational taxonomic units (OTUs). The dominant bacterial genus (*Spiroplasma*) may be an important bacterial factor promoting the occurrence of Chinese cordyceps. For fungi, 289 genera, mainly including *Aureobasidium*, *Candida*, and *Cryptococcus*, were identified, and they belonged to 5 phyla (Ascomycota, Basidiomycota, Chytridiomycota, Glomeromycota, and Zygomycota), 26 classes, 82 orders, and 165 families. Eight bacterial OTUs and 12 fungal OTUs were shared among all of the detected samples and were considered as core species. Among them, *Wolbachia*, *Spiroplasma*, *Carnobacterium*, *Aureobasidium*, and *Phoma* may play important roles in helping the host larva to digest foods, adapt to extreme environments, or resist pathogens. On the other hand, the external (soil) microbial community was synchronously and comparatively analyzed. Comparative analysis revealed that external microbial factors might play a more significant role in the occurrence of Chinese cordyceps, owing to the significant differences revealed by α-diversity and β-diversity analyses among different groups. In summary, the results of this study may contribute to the large-scale cultivation of Chinese cordyceps.

## 1. Introduction

*Ophiocordyceps sinensis* is a well-known entomogenous fungus especially distributed in the Qinghai–Tibetan Plateau and adjacent areas with high altitudes [1,2]. *O. sinensis* obligately parasitizes the soil-borne larva of *Thitarodes* (Hepialidae, Lepidoptera) [3] and ultimately forms fungus–caterpillar complex, which is generally nominated as Chinese cordyceps [1]. In this paper, in order to avoid misunderstanding, we used Chinese cordyceps to refer to the fungus–larva combination and *O. sinensis* to refer to the fungus. Chinese cordyceps have been widely used as a traditional medicinal herb to treat diverse diseases for thousands of years in Oriental countries, particularly in China [4,5].

**Figure 1:**
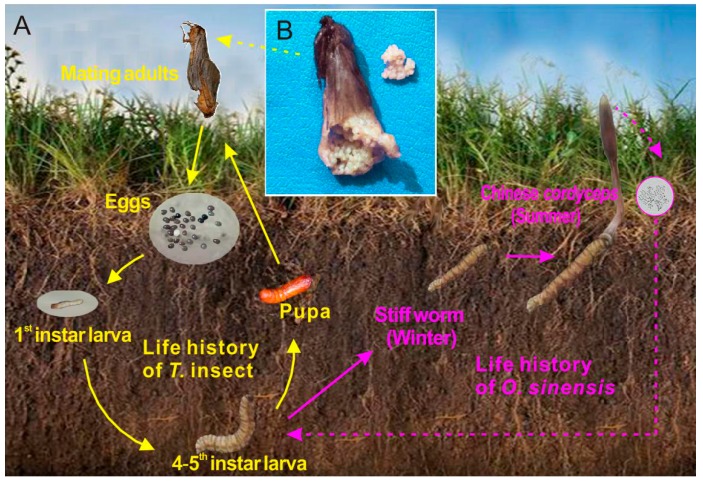
The life cycle of *Ophiocordyceps sinensis* and *Thitarodes* host (**A**) [7], and the unfertilized eggs with attached ovarian tissue from the female *Thitarodes* moth (**B**).

The preeminent pharmaceutical effect induces a great demand of wild Chinese cordyceps [6], whereas the yield is very low owing to its obligate parasitism, complex life history (Figure 1) [7], and eco-geographical preference [8]. What is worse, the yield of wild Chinese cordyceps has sharply decreased in recent years because of excessive excavation, habitat destruction, and climate change [9]. The serious disequilibrium between demand and supply leads to its soaring retail price [10]. Although an increasing number of studies have focused on the large-scale artificial cultivation of Chinese cordyceps, it has not been realized, and many unsolved questions still remain on the relation between *O. sinensis* fungus and its host insects [11,12] (e.g., when and how does the fungi/fungus initially colonize(s) the host larva(e)? What is the crucial factor triggering the occurrence of Chinese cordyceps?)

Generally, infection of the host *Thitarodes* larva mainly happens in soils (Figure 1). The fungal spores of *O. sinensis*, erupted from the mature stroma of Chinese cordyceps, scatter in top soils at random, gradually infiltrate deeper into the soil (mainly caused by rainfall), become infective conidia, and enter into the larva. Besides the fungus *O. sinensis*, our previous study also revealed that the physicochemical factors, the whole microbial structure, and the network of these factors are closely related with the occurrence of Chinese cordyceps, indicating that the soil ecological environment is crucial for its occurrence [13]. We also observed that, although sampled from the same site, the fates of wild larvae varied greatly, and a proportion of these larvae turned into stiff worms and eventually became Chinese cordyceps [14]. Thus, according to these studies, we infer that, besides external soil factors, internal factors (such as the entophytic microbes and immune system of *Thitarodes*) may also be relevant with the infection of *O. sinensis* fungus. Existing researches on symbiotic bacteria and fungi colonized in *Thitarodes* larva are limited to their intestinal microbiota using pure culture or restriction fragment length polymorphism (RFLP) methods [15,16]. Obviously, the above-mentioned target samples in the larva stage are unable to discriminate between stable, colonized internal microbes in the host larva and transient microbes passively transferred from soils and/or foods.

During the early life of *Thitarodes* moths, the eggs of *Thitarodes* inevitably attach to environmental microbiota after being laid out on the grass. In addition, mating behaviors also bring external microbes to the fertilized eggs [17]. Generally, mating behaviors of *Thitarodes* moths immediately occur after the eclosion of pupae. The mating process may averagely last 2.5 h, and the sperm of a male moth are stored in the bursa copulatrix near the ovipositor of the female [17]. After mating, the female moth begins to lay eggs. Fertilization follows ovulation, and the stored sperm are synchronously delivered into the eggs through its micropyles [18]. Owing to these contact opportunities, fertilization may bring various microbes from the bursa copulatrix and spermatheca of female moths as well as male moths into the effluent eggs.

To eliminate contamination from the environment, even including the fertilization process, in this study, we innovatively investigated the bacterial and fungal community of unfertilized *Thitarodes* eggs (Figure 1B) from wild female *Thitarodes* moths via high-throughput sequencing under the premise of excluding the direct interference from habitat soils. The diversity and potential functions of maternally inherited bacteria and fungi, including *Cordyceps*-related fungi, are accordingly discussed. Additionally, the microbial communities of the external soil environment were comparatively analyzed. The results may provide a new clue for further clarifying the relationship between fungi and their host, with special reference to the occurrence of Chinese cordyceps.

## 2. Materials and Methods

### 2.1. Field Site Description and Sample Collection

The native habitats of Chinese cordyceps at Shergyla Mountain, Tibet, were chosen as the study region. Between the years of 2006 and 2016, a pre-survey was carried out via field investigation on the density of Chinese cordyceps and *Thitarodes* larvae [13]. Accordingly, the occurrence rates of Chinese cordyceps in each study region were evaluated. Field investigation showed that the peaks of activity, growth, feeding, development, and population density of *Thitarodes* larvae often appeared between June and August in each year, in particular, around mid-July. According to the field investigation and our previous study [13], three sampling sites were selected. Briefly, site A had a high density of *Thitarodes* larva and Chinese cordyceps (50 larvae/m^2^, 5 Chinese cordyceps/m^2^, occurrence rate 10.0%), site B had a high density of *Thitarodes* larvae and a low density of Chinese cordyceps (70 larvae/m^2^, 1 Chinese cordyceps/m^2^, occurrence rate 1.4%), and site C had a high density of *Thitarodes* larva but had no Chinese cordyceps (75 larva/m^2^, 0 Chinese cordyceps/m^2^, occurrence rate 0). To reduce the difficulties of field sampling for mature eggs, the female moths of *Thitarodes* that were conducting their mating behaviors were sampled. In each sampling site, five pairs of the mating *Thitarodes* moths (identified as *Thitarodes pui*, Figure 1A) were randomly and dispersedly collected in mid-July 2015. The freshly collected moth samples were immediately stored in DNA storage. Three soil samples at each sampling site were collected synchronously. All of the samples were stored inside ice-cold cages and delivered to the laboratory. 

In laboratory, for the moth samples, egg masses (Figure 1B) were carefully taken out from female moths under sterile conditions and named as E-A1 to E-A5, E-B1 to E-B5, and E-C1 to E-C4 (a female moth collected at site C was found without eggs in its ovary), respectively. Owing to the close adhesion between eggs and ovarian tissue, we failed to fully separate the unfertilized eggs from the adhesive ovarian tissue of female moths. The soil samples were sieved through a 2 mm mesh to remove the roots, plant residues, and stones and named as S-A1 to S-A3, S-B1 to S-B3, and S-C1 to S-C3, respectively. After the above procedures, all of the samples were stored at −20 °C before DNA extraction. 

### 2.2. DNA Extraction, PCR, Library Preparation, Sequencing, and Data Analysis

These procedures were performed according to our previous study [13]. Briefly, the MO BIO PowerSoil^®^ DNA Isolation Kit (MO BIO Laboratories Inc., Carlsbad, CA, USA) was employed to extract and purify the total DNA of unfertilized eggs and soil samples according to the manufacturer’s instructions, with sterile water as negative control. The purified DNA was quantified using the NanoDrop ND−3300 spectrophotometer (NanoDrop Technologies, Thermo Scientific, Wilmington, DE, USA). 

Subsequently, the V4 region of bacterial 16S rDNA and the ITS2 region of the fungal ITS gene were specifically amplified using PCR. Primers 515F/806R and ITS3/ITS4, specific for bacterial and fungal fragments, respectively, were used. The primers contained a 12-bp barcode sequence at the 5′-end to distinguish the samples. The PCR reaction mixture (50 μL) contained Ex Taq DNA polymerase (0.5 units; TaKaRa, Dalian, China), 1× Ex Taq loading buffer (10 μL; TaKaRa, Dalian, China), dNTPs (8 μL; TaKaRa, Dalian, China), 2 μL of each primer (10 mM), and DNA template (10–100 ng). PCR was performed by the ABI GeneAmp^®^ 9700 PCR System (Applied Biosystems, Waltham, MA, USA) using the following conditions for bacterial-specific fragments: 95 °C for 3 min; 35 cycles of 94 °C for 30 s, 55 °C for 1 min, and 72 °C for 1 min; and 72 °C for 10 min. For fungal-specific fragments, the PCR procedures included 95 °C for 5 min; 30 cycles of 95 °C for 30 s, 52 °C for 30 s, and 72 °C for 45 s; and final extension at 72 °C for 10 min. Triplicate PCR reactions were carried out for each sample, and the products were mixed. After evaluation by 2% agarose gel electrophoresis, the mixed products were purified with EZNA Gel Extraction Kit (Omega Bio-Tek, Norcross, GA, USA). 

Sequencing libraries were generated using NEBNext^®^ Ultra™ DNA Library Prep Kit for Illumina^®^ (New England Biolabs, MA, USA) following the manufacturer’s recommendations, and index codes were added. The library was assessed on the Qubit 2.0 Fluorometer (Thermo Fisher Scientific, MA, USA) and Agilent Bioanalyzer 2100 system (Agilent Technologies, Waldbron, Germany). Then, it was sequenced on an Illumina MiSeq PE300 platform, and 250 bp paired-end reads were generated (Omics Biotechnology Co., Ltd. Shenzhen, China). To obtain high-quality clean reads, paired-end raw reads were filtered according to the Trimmomatic quality control process [19]. Paired-end clean reads were merged using FLASH according to the relationship of the overlap between the paired-end reads [20]. Sequences were assigned to each sample based on their unique barcode and primer using Mothur software [21]. The obtained sequence data were deposited in the Sequence Read Archive (SRA; SRP136045 and SRP117637). Sequences analysis was performed by Usearch software [22], and sequences with ≥97% similarity were assigned to the same operational taxonomic units (OTUs) [23]. The most frequently occurring sequence was extracted as the representative sequence for each OTU and was screened for further annotation. 

For each representative sequence, the SILVA (for 16S, v. 119; http://www.arb-silva.de) and Unite (for ITS, v. 7.0; http://unite.ut.ee/index.php) databases were used to annotate taxonomic information (set the confidence threshold default to ≥0.5). The OTU and its Tags, which are annotated as chloroplasts or mitochondria (16S amplicons) and cannot be annotated to the kingdom rank, were removed. The OTU taxonomy synthesis information table (OTU table, Tables S1–S4) for the final analysis was generated. 

### 2.3. Data Normalization and Statistical Analysis

Based on the OTU table, Venn diagrams were drawn with R software to illustrate the unique and the shared OTUs among the three groups [24]. The annotation ratio on each classification rank was calculated to obtain the sequence composition of each sample at each classification rank. Based on the relative abundance of species at each classification, R software was used to draw the histogram and heat map.

For α-diversity and β-diversity analyses, OTU tables were rarefied at 11,477 tags (eggs) and 35,000 tags (soil) from 16S rRNA tags per sample, and at 3,664 tags (eggs) and 18,835 tags (soil) from ITS tags per sample. α-diversity was applied in analyzing the complexity of species diversity for a sample through 3 indices, including Chao1, Shannon, and Simpson. All indices in our samples were calculated with QIIME [25]. Chao1 was selected to identify community richness. Shannon and Simpson were used to identify community diversity. β-diversity analysis was used to evaluate differences of samples in species complexity. β-diversity was calculated using weighted UniFrac distance by QIIME software and displayed using principal coordinate analysis (PCoA) by qiime2 and ggplot2 packages in R software [25].

Three non-parametric analyses (analysis of similarity (ANOSIM), non-parametric multivariate analysis of variance (adonis) using distance matrices, and a multiresponse permutation procedure (MRPP)) were performed by R software based on the OTU table to display the extent of differences among groups and whether the differences were significant (*p* < 0.05). A linear discriminant analysis (LDA) effect size (LEfSe) algorithm was employed to identify the taxa in different abundances (biomarker) [26] between the Chinese cordyceps group (sites A and B) and null Chinese cordyceps group (site C). The effect size threshold of the LDA score was set to 2.

## 3. Results

### 3.1. Microbial Diversities 

**Figure 2:**
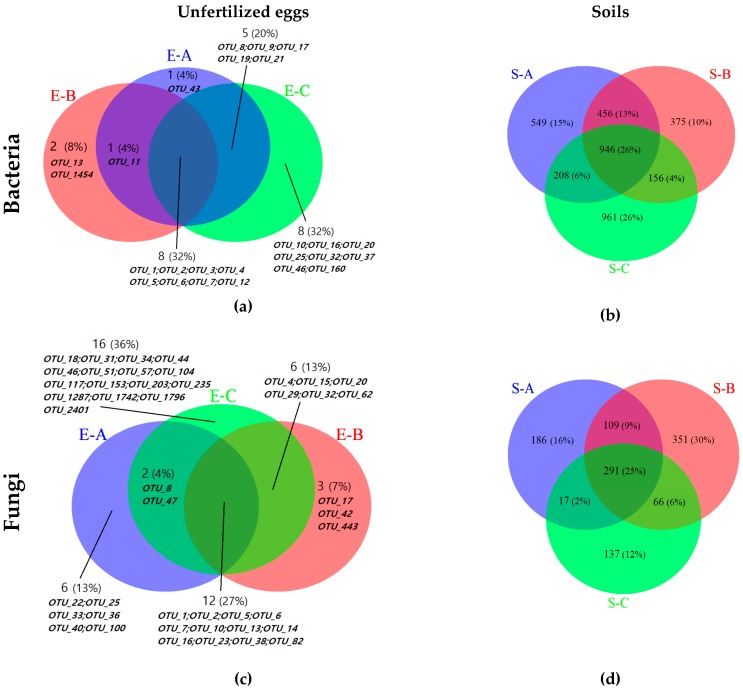
Venn diagrams depicting the unique and shared operational taxonomic units (OTUs) among the samples from different sampling sites. (**a**,**b**) for bacterial community in unfertilized eggs and soil samples, respectively. (**c**,**d**) for fungal community in unfertilized eggs and soil samples, respectively. A, B, and C in each diagram present different sampling sites with high, low, and null Chinese cordyceps, respectively. S-A, S-B, and S-C represent the soil samples from sites A, B and C, respectively; E-A, E-B, and E-C represent the unfertilized eggs samples from sites A, B and C, respectively. The OTUs for each site selected for generating the Venn diagrams were the shared OTUs among all of the samples within each group (e.g., for bacterial OTUs in E-B, 11 OTUs in total were shared among E-B1, E-B2, E-B3, E-B4, and E-B5, and these 11 OTUs were selected to generate the Venn diagram). For unfertilized eggs samples, bacterial OTU_1 (*Wolbachia*), OTU_2 (Firmicutes), OTU_3 (Gammaproteobacteria), OTU_4 (*Spiroplasma*), OTU_5 (*Carnobacterium*), OTU_6 (*Acinetobacter*), OTU_7 (*Sphingobium*), and OTU_12 (*Cupriavidus*) are shared among all of the egg samples; fungal OTU_1 (Basidiomycota), OTU_2 (Basidiomycota), OTU_5 (*Aureobasidium*), OTU_6 (Sordariomycetes), OTU_7 (Fungi), OTU_10 (Fungi), OTU_13 (Fungi), OTU_14 (*Phoma*), OTU_16 (Fungi), OTU_23 (Basidiomycota), OTU_38 (Agaricomycetes), OTU_82 (*Davidiella*) are shared among all of the egg samples. The detailed taxonomic information of the OTUs were supplemented in Tables S1–S4.

The number of high-quality sequences and OTUs are shown in Table A1 and Table 1, and the α-diversity indices of bacterial and fungal communities are shown in Table 1. For unfertilized egg samples, there were totals of 373,511 high-quality 16S rDNA sequences and 601,971 high-quality ITS2 sequences. From these high-quality sequences, 2213 bacterial OTUs (Table 1 and Table S1) and 2452 fungal OTUs (Table 1 and Table S2) were clustered, respectively, with a 97% identity threshold [23]. The bacterial diversity (represented by Shannon index and Simpson index) of site C (null Chinese cordyceps group) was significantly higher than that of site B (low Chinese cordyceps group, *p* < 0.05). For soil samples, there were totals of 477,833 high-quality 16S rDNA sequences and 317,533 high-quality ITS2 sequences. From this, 26,835 bacterial OTUs (Table 1 and Table S3) and 9219 fungal OTUs (Table 1 and Table S4) were clustered. Notably, all of the α-diversity indices of soil were significantly higher than those of the unfertilized egg samples. Venn diagrams showed the proportions of the unique and shared OTUs in eggs (Figure 2a,c) or soils (Figure 2b,d) among the three sampling sites. The proportion of overlap represented the number of shared OTUs and were considered as the core microbiome [24]. Bacterial OTU_1 (*Wolbachia*), OTU_2 (Firmicutes), OTU_3 (Gammaproteobacteria), OTU_4 (*Spiroplasma*), OTU_5 (*Carnobacterium*), OTU_6 (*Acinetobacter*), OTU_7 (*Sphingobium*), and OTU_12 (*Cupriavidus*) and fungal OTU_1 (Basidiomycota), OTU_2 (Basidiomycota), OTU_5 (*Aureobasidium pullulans*), OTU_6 (Sordariomycetes), OTU_7 (Fungi), OTU_10 (Fungi), OTU_13 (Fungi), OTU_14 (*Phoma*), OTU_16 (Fungi), OTU_23 (Basidiomycota), OTU_38 (Agaricomycetes), and OTU_82 (*Davidiella*) were shared among all of the detected eggs samples and were considered as core species. For bacterial OTUs, 1 OTU, 2 OTUs and 8 OTUs were unique in unfertilized eggs samples from site A, B and C, respectively. For fungal OTUs, 6 OTUs, 3 OTUs and 16 OTUs were unique in unfertilized eggs samples from site A, B and C, respectively. For soil samples, 946 bacterial OTUs and 291 fungal OTUs were shared among all of the sampling sites.

**Figure 3:**
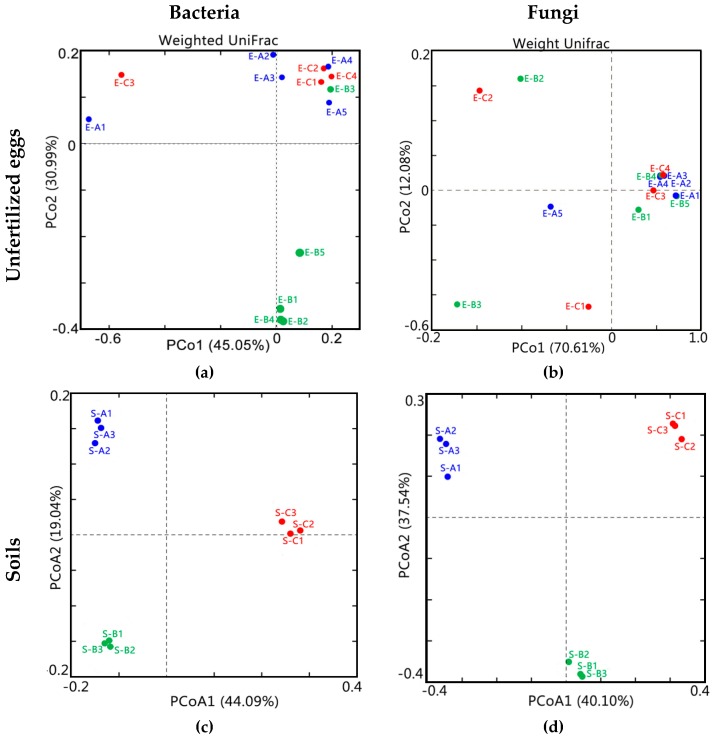
PCoA plots based on Weighted Unifrac distances of OTU profiles of unfertilized eggs (**a**,**b**) and soil (**c**,**d**) samples. A, B, and C present different sampling sites with high, low, and null Chinese cordyceps, respectively. Blue, green, and red circles represent samples from sites A, B, and C, respectively.

To evaluate the β-diversity changes in unfertilized eggs and soils across different sites, a principal coordinate analysis (PCoA) was applied based on the weighted Unifrac distance matrixes. For unfertilized egg samples, the bacterial (Figure 3a) and fungal (Figure 3b) β-diversities were not different in each site, and distances between the samples within each site were not close. Corresponding to the ANOSIM, Adonis, and MRPP analyses (Table 2), significant differences were proven (*p* < 0.05) among the unfertilized egg samples of three sites from a bacterial community aspect, while no significant differences were observed (*p* > 0.05) from a fungal community aspect. Thus, the bacterial communities of the unfertilized egg samples were significantly different among different sampling sites. For soil samples, the microbiota could be separated into three groups. The bacterial (Figure 3c) and fungal (Figure 3d) β-diversities were significantly different for each site, and the significant differences were proven (*p* < 0.05) among all of the soil samples from bacterial and fungal community aspects, as shown in Table 2.

### 3.2. Bacterial and Fungal Structure

The relative compositions of bacterial and fungal communities at phylum, class, order, family, and genus ranks are presented in Tables S1–S4. For unfertilized egg samples, among the 1294 bacterial OTUs, 1180 OTUs (91.9%) were accurate at least to the phylum rank, 1125 OTUs (86.94%) to the class rank, 1007 OTUs (77.82%) to the order rank, 774 OTUs (59.81%) to the family rank, and 618 OTUs (47.76%) to the genus rank (Table S1); for all 907 fungal OTUs, 531 OTUs (58.54%) were accurate at least to the phylum rank, 347 OTUs (38.26%) to the class rank, 277 OTUs (30.54%) to the order rank, 212 OTUs (23.37%) to the family rank, and 153 OTUs (16.87%) to the genus rank (Table S2). For soil samples, among the 26,835 bacterial OTUs, 26,784 OTUs (99.81%) were accurate at least to the phylum rank, 26,245 OTUs (97.80%) to the class rank, 22,758 OTUs (84.81%) to the order rank, 15,136 OTUs (56.40%) to the family rank, and 5654 OTUs (21.07%) to the genus rank (Table S3); among the 9214 fungal OTUs, 7549 OTUs (81.93%) were accurate at least to the phylum rank, 6440 OTUs (69.89%) to the class rank, 5924 OTUs (64.29%) to the order rank, 5221 OTUs (56.67%) to the family rank, and 4009 OTUs (43.51%) to the genus rank (Table S4). 

**Figure 4:**
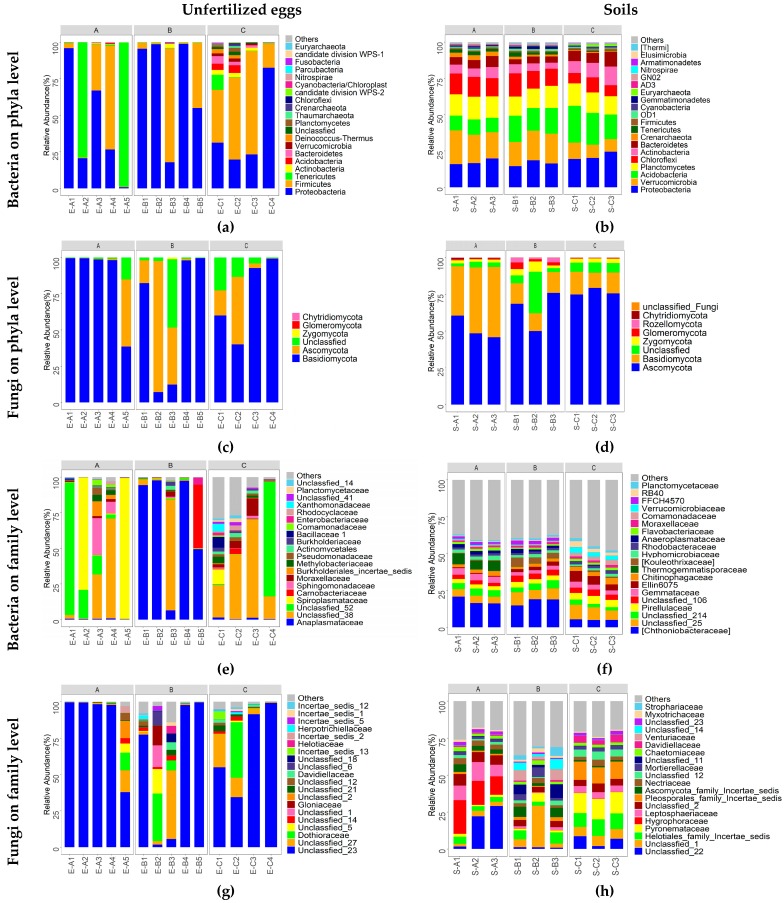
The relative abundances of the top 20 microbial phyla and families in unfertilized eggs (**a**,**c**,**e**,**g**) and soil (**b**,**d**,**f**,**h**) samples at different sites. A, B, and C present different sampling sites with high, low, and null Chinese cordyceps, respectively. Detailed taxa information is presented in Table A2, Table A3, Table A4 and Table A5; “Others” include phyla or families beyond the top 20 phyla.

Figure 4 intuitively illustrates the top 20 phyla and top 20 families of bacterial and fungal communities. At phylum rank (Figure 4a–d), the microbial structures displayed significantly different patterns between unfertilized eggs and soil samples. For unfertilized egg samples, both the predominant phyla of bacterial communities and fungal communities varied among different individual samples and did not present remarkable differences among different groups. For the bacterial community (Figure 4a), Proteobacteria, Firmicutes, and Tenericutes were the most predominant in E-A1, E-A3, E-B1, E-B2, E-B4, E-B5, and E-C4; E-A4, E-B3, E-C1, E-C2, and E-C3; and E-A2 and E-A5, respectively. For the fungal community (Figure 4c), Basidiomycota was the predominant fungal phylum in 10 unfertilized eggs samples (E-A1 to E-A4, E-B1, E-B4, E-B5, E-C1, E-C3, and E-C4), and in the remaining four unfertilized egg samples (E-A5, E-B2, E-B3, and E-C5), Ascomycota was overwhelmingly predominant, followed by Basidiomycota. For soil samples, the predominant bacterial (Figure 4b) and fungal (Figure 4d) phyla were distributed stably and evenly among different samples.

At family rank (Figure 4e–h and Table A2, Table A3, Table A4 and Table A5), for unfertilized egg samples, both the predominant family of bacterial communities (Figure 4e) and fungal communities (Figure 4g) varied among different individual samples and did not present remarkable differences among different groups. For bacteria (Figure 4e), Anaplasmataceae was predominant in E-B1, E-B2, E-B3, and E-B5 with a relative abundance range of 49.5–97.4%. Unclassfied_38 (Firmicutes) ranked first in E-A3, E-A4; E-B3; and E-C1, E-C2, and E-C3. Unclassfied_52 (Proteobacteria; Gammaproteobacteria) and Spiroplasmataceae ranked first in E-A1, E-C4 and E-A2, E-A5, respectively. For fungi (Figure 4g), Unclassified_23 (Basidiomycota) ranked first in most samples, except E-B2 and E-B3; Dothioraceae and Unclassified_27 ranked the first in E-B2 and E-B3, respectively. For soil samples, the predominant bacterial families (Figure 4f) were distributed stably and evenly among all samples, and the most abundant bacterial families were in the order of Chthoniobacteraceae, Unclassified_25 (Acidobacteria), Unclassified_214 (Acidobacteria), and Pirellulaceae. The predominant fungal families (Figure 4h) were distributed stably and evenly within sampling sites A and C, while they varied between these two sites. For instance, Unclassified_22 (Basidiomycota), Hygrophoraceae, and Pyronemataceae were the most predominant in sampling site A; Pyronemataceae, Helotiales, and Pleosporales were the most predominant in sampling site C. While for sampling site B, the predominant fungal families varied among different samples.

**Figure 5:**
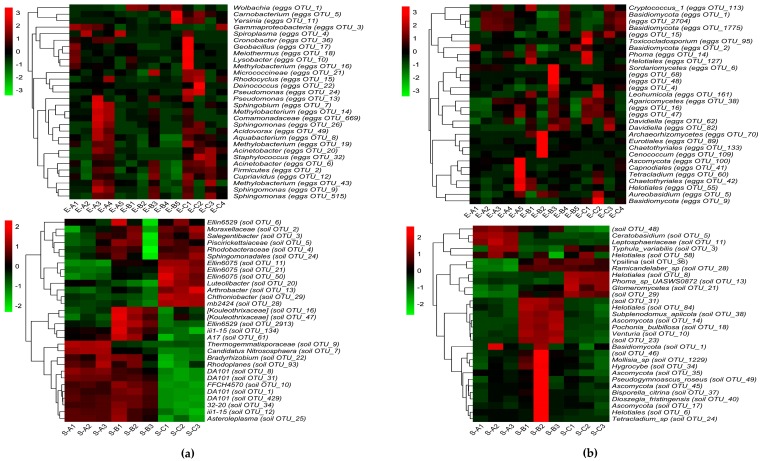
Heatmaps of the top 30 OTUs of bacterial (**a**) and fungal (**b**) communities of unfertilized eggs (E) and soil (S) samples at each site. Relative abundance of the microbial community from high to low is represented by red, black, and green. A, B, and C present different sampling sites with high, low, and null Chinese cordyceps, respectively.

Figure 5 displays the 30 most abundant OTUs of bacterial (Figure 5a) and fungal (Figure 5b) communities in unfertilized eggs and soil samples, respectively, and the relative abundances of microbial community from high to low are represented by red, black, and green. In Figure 5, the top bacterial and fungal genera in unfertilized egg samples were not significantly different among the samples from the three sites. For bacteria, OTU1 (Proteobacteria; Alphaproteobacteria; Rickettsiales; Anaplasmataceae; *Wolbachia*) was the highest in site B (E-B1, E-B2, E-B4, and E-B5); OTU2 (Firmicutes) was the highest in E-A5, E-A2, E-B4, E-B2, E-B1, and E-B5. OTU3 (Proteobacteria; Gammaproteobacteria) was the highest in E-A1, E-A2, E-A3, and E-C4, and OTU4 (Tenericutes; Mollicutes; Entomoplasmatales; Spiroplasmataceae; *Spiroplasma*) was the highest in E-A2, E-A5, and E-C1. For fungi, OTU1 (Basidiomycota) was the highest in E-A2, E-A3, E-A4, E-A5, E-B4, E-C3, and E-C4; OTU2 (Basidiomycota) was the highest in E-A1, E-B1, E-B5, and E-C1; OTU5 (Ascomycota; Dothideomycetes; Dothideales; Dothioraceae; *Aureobasidium*) was the highest in E-B2 and E-C2. Distinct with the unfertilized egg samples, the top bacterial and fungal genera in soil samples were evenly distributed and presented different heatmap patterns among different sites. In addition, the taxonomic information of those OTUs revealed the distinct microbial community between egg and soil samples, owing to that they did not share the same OTUs. 

### 3.3. Differential OTUs Related with the Occurrence of Chinese cordyceps

**Figure 6:**
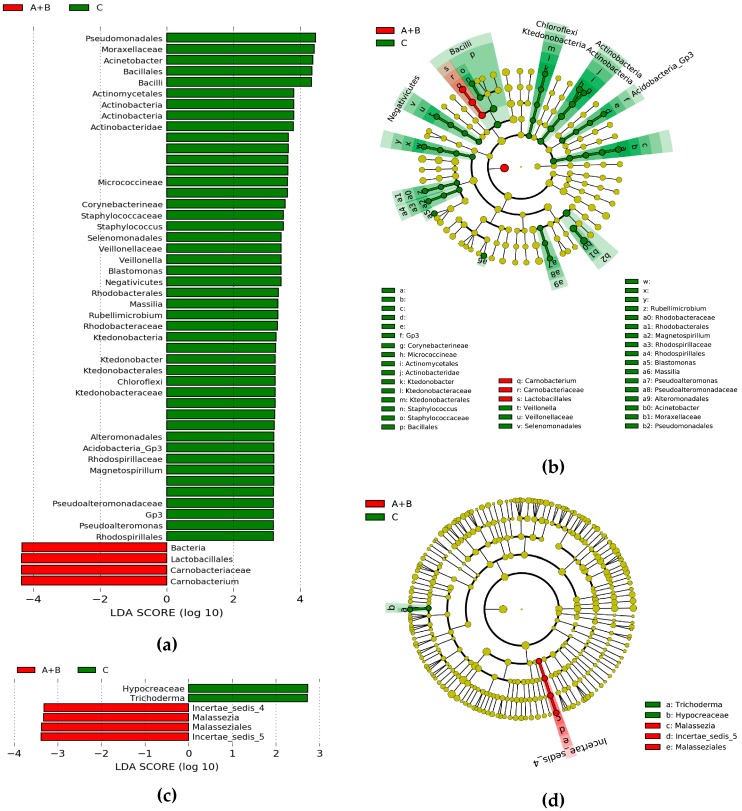
The microbial taxa with different abundances in Chinese cordyceps groups (sites A and B) and null Chinese cordyceps group (site C) illustrated using linear discriminant analysis (LDA) effect size analysis and cladograms. (**a**,**c**), the LDA scores of biomarkers of bacteria and fungi illustrated by histograms, respectively. Microbial taxa were analyzed by LDA with a significant threshold over 2.0, and the length of each bar represents the degree of differences. (**b**,**d**), the taxonomic information of these bacterial and fungal biomarkers was illustrated by cladograms, respectively, with circles radiating from the center point representing the taxonomic ranks from phylum to species. Red bars or nodes, taxa enriched in sites A and B; Green bars or nodes, taxa enriched in site C. Yellow nodes, taxa with no significant difference.

In order to discuss the detailed OTUs colonized in the *Thitarodes* host, which might be related to the occurrence of Chinese cordyceps, the differential OTUs between Chinese cordyceps groups (sites A and B) and null Chinese cordyceps group (site C) were screened using linear discriminant analysis (LDA) effect size analysis and illustrated by histograms (Figure 6a,c) and cladograms (Figure 6b,d). Four bacterial OTUs (mostly belonging to the class of Lactobacillales) and four fungal OTUs (mostly belonging to the class of Malasseziales) presented significantly higher abundances in the egg samples of Chinese cordyceps groups (sites A and B). And 46 bacterial OTUs and 2 fungal OTUs (mostly belonging to the family of Hypocreaceae) were significantly enriched in the null Chinese cordyceps group (site C). 

## 4. Discussion

### 4.1. The Internal Microbial Community is Significantly Different from that in the External Soil Environment 

High-throughput sequencing of the *Thitarodes* unfertilized eggs revealed highly diverse colonized bacterial and fungal communities. *Thitarodes* is a holometabolous insect with four developmental stages, i.e., egg (30–40 days), larva (3–4 years or even 4–5 years), pupa (approximately 40 days), and moth (3–8 days) (Figure 1) [11]. Among these stages, the larva is the only feeding stage, and various microbes from soils and foods may enter the larva via its feeding behaviors. Until recently, the studies on the microbial composition in the *Thitarodes* were focused on the intestinal fungi [15] or bacteria [16] of larva, which could not discriminate microbes that were stably colonized in their host and the transient external environmental soil microbes through feeding behaviors. The relation between *Thitarodes* and its symbiotic fungi or bacteria is still a pending crucial problem. For the moth stage, *Thitarodes* does not intake food for longer than a month. It can be inferred that this stage could filter out parts of the intestinal microbes that could not be colonized in the internal environment of the *Thitarodes*, or the microbes were cleared out by the immune system of *Thitarodes*. This study comparatively analyzed the microbial composition of the internal and external environments. The results revealed a highly diverse fungal and bacterial community (907 fungal OTUs and 1294 bacterial OTUs), and a total of 150 fungal genera and 346 bacterial genera were identified in the unfertilized eggs of female moths. However, the microbial composition (Figure 4, Figure 5 and Figure 6) in the eggs presented evident difference with that of the soil environment. Thus, the internal environment may reshape the colonized microbial community.

The microbial community in soil presented a close relation with the sampling sites. While different from soil samples, both α- and β-fungal diversities in the *Thitarodes* unfertilized eggs varied irregularly among different samples, both for intergroup or intragroup cases (Table 1 and Figure 3a,b). The data revealed that the differences of the soil microbial community among different sampling sites (with different occurrence rates of Chinese cordyceps) were significant, while the microbial composition varied irregularly for the unfertilized eggs within or without each sampling site. Thus, it can be inferred that external microbial factors might contribute more to the occurrence of Chinese cordyceps [13]. 

### 4.2. Internal Microbial Composition in the Unfertilized Eggs of Thitarodes 

The co-evolution between insect and fungi or bacteria is a common phenomenon in nature [27,28]. Having inhabited the high and cold alpine regions for thousands of years, *Thitarodes* in the larva stage in soils prefers to feed on the tender roots of plants, which contain a high proportion of indigestible cellulose [29]. Furthermore, the larva generally undergoes durations of food shortages in winter [29,30,31]. It has been proven that the host larva may not produce cellulase and hemicellulase, but it can utilize some colonized microbes to aid in digestion [32]. In addition, several entomopathogens of *Thitarodes* (e.g., *Beauveria bassiana*) are endowed with mannitol-1-phosphate dehydrogenase and mannitol dehydrogenase, which help the host to tolerate stressors such as UV radiation and soaring heat in the Tibetan Plateau via regulating the accumulation of mannitol [33]. 

Identifying the core microbiome is essential to unravel the ecology of microbial consortia because it has been proposed that the commonly occurring organisms that appear in all assemblages associated with a particular habitat are likely critical to the functions of that type of community [24]. In this study, for unfertilized *Thitarodes* eggs, 8 bacterial OTUs and 12 fungal OTUs were shared among all of the detected samples and were considered as core species. These microbes might be important for *Thitarodes* host. Among them, *Wolbachia* and *Spiroplasma* have evolved the ability to cause reproductive alterations in their arthropod hosts, such as cytoplasmic incompatibility (CI), parthenogenesis, feminization, and male killing [34], and might modulate immune signaling against infection by certain insect pathogenic and non-pathogenic bacteria [35]; *Carnobacterium*, which is the dominant bacteria in the intestine of *Thitarodes*, can promote the growth of *Thitarodes* larvae, elevate bacterial diversity, maintain a better balance of intestinal flora, and act as a probiotic in *Thitarodes* [36]; *Aureobasidium* is an overwhelmingly dominant fungus, which has anti-microbial activities and is also capable of producing useful enzymes, such as amylases, cellulases, lipases, proteases, xylanases, and mannanases [37]; *Phoma* is proven to have an ability to produce antibiotics and economically useful secondary metabolites [38]. Besides those core microbes, some predominant (30 most abundant) bacterial or fungal OTUs might also be important to *Thitarodes* host. Among the predominant bacterial and fungal OTUs (Figure 5), *Stenotrophomonas* is able to digest carboxymethylcellulose [39]; *Cryptococcus*, with an antagonistic activity against filamentous fungi, is one yeast species isolated from the natural nests of *Atta texana* [40]. Therefore, as indigenous organisms inhabited in the Qinghai–Tibet Plateau for thousands of years, those microbes identified in this study may play important roles in aiding the host larva to survive. Reciprocally, the host larvae provide preferable shelters to these indigenous microbes for maintaining their diversity. 

### 4.3. Discovery of Cordyceps-Related Fungi in the Unfertilized Eggs of Thitarodes

*Cordyceps*-related fungi may enter the host *Thitarodes* as early as in the oocyte stage. This perception provides a novel clue for studying infection mechanisms. Owing to the existence of the tough surface cuticle (composed of wax and epicuticle) of *Thitarodes* larvae, the fungi related to Chinese cordyceps undergo a tough entrance into the cuticle. It is generally considered that fungi enter their host by the mouth and make their way through the gut or ecdysis behavior in the larva stage in soils [41,42]. However, no sufficient evidence has been provided to validate this claim [11]. Previous studies suggested that the yeast-like symbiont, an uncultured fungal endosymbiont belonging to the Ophiocordycipitaceae family in arthropods [43], might occur in the fat body of its host and then be transmitted to its offspring through the ovary. In this case, *Purpureocillium*, which also belongs to the Ophiocordycipitaceae family [44], was detected in unfertilized egg samples from the *Cordyceps* group (site A and site B, Table S2), and it might be also transmitted into the *Thitarodes* offspring via the ovary. The discovery of the Ophiocordycipitaceae family from the unfertilized eggs of *Thitarodes* might provide the possibility of maternal infection and enlarge the knowledge on the infection mechanisms, while the speculation should be tested and confirmed by further experiments. 

Microbial diversity analysis suggests that there was no significant difference among the sample groups with different occurrence rates of Chinese cordyceps. This means that there is no clear linkage between the colonized microbial diversity in the unfertilized egg stage of *Thitarodes* and the occurrence rate of Chinese cordyceps. Therefore, microbial contribution to the occurrence of Chinese cordyceps may be generated from the subsequent life cycle of *Thitarodes*. Although the overall microbial diversity presents no significant difference among different sample groups, several identified genera may provide new knowledge on infection mechanisms. This study revealed that 46 bacterial and 2 fungal OTUs were significantly enriched in the null Chinese cordyceps group (site C) based on LDA (with LDA scores higher than 2, Figure 6). Among them, *Actinomycetes* can produce various antibiotics [45]; *Acinetobacter* (a symbiont isolated from the insect gut) may contribute to de-novo purine biosynthesis and its metabolism [46]; *Trichoderma asperellum*, with the abilities of arsenic resistance and arsenic speciation transformation [47], may assist the host to detoxify the toxic arsenic accumulated along the trophic chain from the high arsenic background in Tibetan soils [18.7 mg/kg; evidently higher than the average abundance of the upper continental crust (1.5 mg/kg)] [48]. Thus, the enriched microbes at site C might benefit the *Thitarodes* moths and indirectly suppress the occurrence of Chinese cordyceps. Additionally, we noticed that fungal genus *Malassezia*, which was generally associated with the skins of mammalian hosts, was specially enriched in the egg samples of Chinese cordyceps group (sites A and B). Recently, *Malassezia* was frequently discovered (deep sea and saline alkali soil) and proved exceedingly widespread and ecologically diverse [49,50]. This study enlarged the knowledge on the coverage of *Malassezia*, while the role of the enriched *Malassezia* in Chinese cordyceps group (sites A and B) on the occurrence should be clarified in future. Conclusively, although the microbial diversity and community presented no significant relation with the occurrence rate of Chinese cordyceps, these screened differential microbes might play roles in the occurrence and the roles should be validated by future study.

Besides the above screened differential genera, *Spiroplasma* was one of the most abundant annotated genera (Figure 5). The abundance of *Spiroplasma* was mostly represented by site A, especially by samples A2 and A5, the relative abundances of which were approximately 98.6% and 78.8%, respectively. *Spiroplasma* are considered to be pathogens and are widely thought to be male-killing biofactors in insects [51]. Coincidently, several abnormal behaviors of the infected *Thitarodes* moths at the symptomatic stage caused by *Spiroplasma* were observed in other arthropods [52,53]. Therefore, in this study, we speculate that the occurrence of Chinese cordyceps may be aided by *Spiroplasma*, which is the dominant bacterial genus in the unfertilized eggs of female *Thitarodes* moths. However, this hypothesis was merely based on the coincidences and correlation between the abundance of *Spiroplasma* and the potential occurrence of Chinese cordyceps. Experiments on validating *Spiroplasma* infection cannot be easily performed because of its endosymbiotic characteristics. Thus, further validations should be founded in studies with larger sample populations.

## Figures and Tables

**Table 1:** The number of high-quality sequences, OTUs and α-diversity indices of microbial communities in unfertilized eggs and soils of different sampling sites.

Classified	Sample Sites	Number of Sequences	Number of OTUs	Shannon	Simpson	Chao1
Bacteria	Egg A	27,345 ± 4854	94 ± 35	1.47 ± 1.34	0.37 ± 0.32	112.25 ± 39.36
	Egg B	25,325 ± 5290	61 ± 23	0.78 ± 0.66	0.23 ± 0.23	86.54 ± 14.91
	Egg C	27,541 ± 6983	360 ± 538	3.35 ± 2.19 #	0.64 ± 0.27 #	382.7 ± 561.93
	Total	373,511	2213	/	/	/
	Soil A	55,662 ± 3100	2981 ± 80	8.83 ± 0.13	0.99 ± 0.00	3437.59 ± 64.92
	Soil B	51,893 ± 15,637	2864 ± 308	8.52 ± 0.01 *	0.99 ± 0.00 *	3413.66 ± 60.17
	Soil C	51,723 ± 990	3100 ± 24	9.25 ± 0.07 *,#	1 ± 0.00 *,#	3586.21 ± 14.8 *,#
	Total	477,833	26,835	/	/	/
Fungi	Egg A	54,684 ± 38,146	168 ± 28	0.91 ± 1.41	0.21 ± 0.35	39.2 ± 33.75
	Egg B	34,443 ± 32,753	162 ± 54	2.22 ± 1.95	0.48 ± 0.42	94.6 ± 72.78
	Egg C	39,083 ± 34,275	200 ± 30	1.93 ± 1.6	0.43 ± 0.37	82.5 ± 25.75
	Total	601,971	2452	/	/	/
	Soil A	25,308 ± 6655	952 ± 54	6.1 ± 0.47	0.92 ± 0.02	1229.76 ± 41.22
	Soil B	56,914 ± 32,119	1298 ± 270	6.97 ± 0.58	0.96 ± 0.03	1312.59 ± 46.79
	Soil C	23,623 ± 5542	846 ± 59 #	6.55 ± 0.14	0.96 ± 0.00 *	1133.13 ± 18.88 *,#
	Total	317,533	9219	/	/	/

* *p* < 0.05 compared to site A; # *p* < 0.05 compared to site B. Number of sequences and number of OTUs were calculated and presented with average ± SD according to the original data of Table A1. A, B, and C present different sampling sites with high, low, and null Chinese cordyceps, respectively.

**Table 2:** Dissimilarity comparison of unfertilized eggs/soil microbial community structures among the three sampling sites.

	ANOSIM *		Adonis			MRPP			
	*R*	*P*	*F*	*R* ^2^	*P*	Observed Delta (δ)	Expected Delta (δ)	Effect Size (A)	*P*
Bacteria for unfertilized eggs	0.5112	0.003	3.1266	0.3624	0.005	0.6818	0.8126	0.1609	0.013
Bacteria for soils	1.0000	0.003	23.117	0.8851	0.009	0.1940	0.4793	0.5952	0.004
Fungi for unfertilized eggs	−0.1053	0.807	0.78622	0.1251	0.639	0.8316	0.8074	−0.0299	0.690
Fungi for soils	1.0000	0.009	11.057	0.7866	0.003	0.3285	0.6432	0.4893	0.005

* ANOSIM: *R* value approaching 1 indicates that the difference between groups is larger than the difference within groups, while an *R* value approaching 0 indicates that there is no significant difference between groups and within groups. Adonis: *F* represents *F* test value, and *R*^2^ represents the interpretation degree of sample difference by different groups. The larger *F* and *R*^2^ are, the higher the degree of group difference is. *p* < 0.05 indicates that the test has a high feasibility. MRPP: A > 0 indicates that the difference between groups is greater than the difference within groups, and A < 0 indicates that the difference within groups is greater than the difference between groups. Observed Delta and expected Delta indicates the differences within and between the groups, respectively; the larger the Delta value is, the larger the difference is within or between the groups. *p* < 0.05 indicates significant differences among the groups.

## Supplementary Materials

Supplementary materials can be found at https://www.mdpi.com/2076-2607/7/11/517/s1.

## Appendix A

**Table A1:** The number of the obtained high-quality sequences and the corresponding OTU number from each sample.

Sample ID	Number of Sequences (ITS)	Number of OTUs (ITS)	Number of Sequences (16S)	Number of OTUs (16S)
E-A1	114,096	199	25,063	78
E-A2	63,095	149	32,326	60
E-A3	49,102	190	28,918	139
E-A4	34,788	133	20,079	123
E-A5	12,341	169	30,337	68
E-B1	24,247	235	30,383	90
E-B2	3664	87	22,309	43
E-B3	6392	152	22,996	81
E-B4	63,019	184	19,477	42
E-B5	74,894	153	31,458	49
E-C1	15,188	215	36,614	1167
E-C2	17,619	203	22,164	106
E-C3	34,708	157	29,450	80
E-C4	88,818	226	21,937	87
S-A1	18,835	916	53,307	2891
S-A2	32,131	1014	54,504	3045
S-A3	24,957	926	59,174	3008
S-B1	40,748	1224	66,007	3135
S-B2	93,904	1489	54,588	2928
S-B3	36,089	1107	35,083	2529
S-C1	29,793	908	50,667	3110
S-C2	22,007	839	52,630	3072
S-C3	19,069	791	51,873	3117

**Table A2:** The detailed taxonomic information of the top 20 bacteria of unfertilized eggs on family rank.

Ranked List	Annotations in Figure 4e	Detailed Taxonomic Information
1st	Anaplasmataceae	Proteobacteria; Alphaproteobacteria; Rickettsiales; Anaplasmataceae
2nd	Unclassfied_38	Firmicutes; Unclassfied; Unclassfied; Unclassfied
3rd	Unclassfied_52	Proteobacteria; Gammaproteobacteria; Unclassfied; Unclassfied
4th	Spiroplasmataceae	Tenericutes; Mollicutes; Entomoplasmatales; Spiroplasmataceae
5th	Carnobacteriaceae	Firmicutes; Bacilli; Lactobacillales; Carnobacteriaceae
6th	Sphingomonadaceae	Proteobacteria; Alphaproteobacteria; Sphingomonadales; Sphingomonadaceae
7th	Moraxellaceae	Proteobacteria; Gammaproteobacteria; Pseudomonadales; Moraxellaceae
8th	Burkholderiales_incertae_sedis	Proteobacteria; Betaproteobacteria; Burkholderiales; Burkholderiales_incertae_sedis
9th	Methylobacteriaceae	Proteobacteria; Alphaproteobacteria; Rhizobiales; Methylobacteriaceae
10th	Pseudomonadaceae	Proteobacteria; Gammaproteobacteria; Pseudomonadales; Pseudomonadaceae
11th	Actinomycetales	Actinobacteria; Actinobacteria; Actinobacteridae; Actinomycetales
12th	Burkholderiaceae	Proteobacteria; Betaproteobacteria; Burkholderiales; Burkholderiaceae
13th	Bacillaceae 1	Bacilli; Bacillales; Bacillaceae 1
14th	Comamonadaceae	Proteobacteria; Betaproteobacteria; Burkholderiales; Comamonadaceae
15th	Enterobacteriaceae	Proteobacteria; Gammaproteobacteria; Enterobacteriales; Enterobacteriaceae
16th	Rhodocyclaceae	Proteobacteria; Betaproteobacteria; Rhodocyclales; Rhodocyclaceae
17th	Xanthomonadaceae	Proteobacteria; Gammaproteobacteria; Xanthomonadales; Xanthomonadaceae
18th	Unclassfied_41	Unclassfied; Unclassfied; Unclassfied; Unclassfied
19th	Planctomycetaceae	Planctomycetes; Planctomycetia; Planctomycetales; Planctomycetaceae
20th	Unclassfied_14	Acidobacteria; Acidobacteria_Gp4; Gp4; Unclassfied
	Others	Others

**Table A3:** The detailed taxonomic information of the top 20 bacteria of soil samples on family rank.

Ranked List	Annotations in Figure 4f	Detailed Taxonomic Information
1st	[Chthoniobacteraceae]	Verrucomicrobia; [Spartobacteria]; [Chthoniobacterales]; [Chthoniobacteraceae]
2nd	Unclassfied_25	Acidobacteria; Acidobacteria-6; iii1-15; Unclassfied
3rd	Unclassfied_214	Planctomycetes; Phycisphaerae; WD2101; Unclassfied
4th	Pirellulaceae	Planctomycetes; Planctomycetia; Pirellulales; Pirellulaceae
5th	Unclassfied_106	Chloroflexi; Ellin6529; Unclassfied; Unclassfied
6th	Gemmataceae	Planctomycetes; Planctomycetia; Gemmatales; Gemmataceae
7th	Ellin6075	Acidobacteria; [Chloracidobacteria]; RB41; Ellin6075
8th	Chitinophagaceae	Bacteroidetes; [Saprospirae]; [Saprospirales]; Chitinophagaceae
9th	Thermogemmatisporaceae	Chloroflexi; Ktedonobacteria; Thermogemmatisporales; Thermogemmatisporaceae
10th	[Kouleothrixaceae]	Chloroflexi; Chloroflexi; [Roseiflexales]; [Kouleothrixaceae]
11th	Hyphomicrobiaceae	Proteobacteria; Alphaproteobacteria; Rhizobiales; Hyphomicrobiaceae
12th	Rhodobacteraceae	Proteobacteria; Alphaproteobacteria; Rhodobacterales; Rhodobacteraceae
13th	Anaeroplasmataceae	Tenericutes; Mollicutes; Anaeroplasmatales; Anaeroplasmataceae
14th	Flavobacteriaceae	Bacteroidetes; Flavobacteriia; Flavobacteriales; Flavobacteriaceae
15th	Moraxellaceae	Proteobacteria; Gammaproteobacteria; Pseudomonadales; Moraxellaceae
16th	Comamonadaceae	Proteobacteria; Betaproteobacteria; Burkholderiales; Comamonadaceae
17th	Verrucomicrobiaceae	Verrucomicrobia; Verrucomicrobiae; Verrucomicrobiales; Verrucomicrobiaceae
18th	FFCH4570	Chloroflexi; TK10; B07_WMSP1; FFCH4570
19th	RB40	Acidobacteria; Acidobacteria-6; iii1-15; RB40
20th	Planctomycetaceae	Planctomycetes; Planctomycetia; Planctomycetales; Planctomycetaceae
	Others	Others

**Table A4:** The detailed taxonomic information of the top 20 fungi of unfertilized eggs samples on family rank.

Ranked List	Annotations in Figure 4f	Detailed Taxonomic Information
1st	Unclassfied_23	Basidiomycota; Unclassfied; Unclassfied; Unclassfied
2nd	Unclassfied_27	Unclassfied; Unclassfied; Unclassfied; Unclassfied
3rd	Dothioraceae	Ascomycota; Dothideomycetes; Dothideales; Dothioraceae
4th	Unclassfied_5	Ascomycota; Eurotiomycetes; Chaetothyriales; Unclassfied
5th	Unclassfied_14	Ascomycota; Unclassfied; Unclassfied; Unclassfied
6th	Unclassfied_1	Ascomycota; Archaeorhizomycetes; Unclassfied; Unclassfied
7th	Gloniaceae	Ascomycota; Dothideomycetes; Hysteriales; Gloniaceae
8th	Unclassfied_2	Ascomycota; Dothideomycetes; Capnodiales; Unclassfied
9th	Unclassfied_21	Basidiomycota; Agaricomycetes; Unclassfied; Unclassfied
10th	Unclassfied_12	Ascomycota; Leotiomycetes; Helotiales; Unclassfied
11th	Davidiellaceae	Ascomycota; Dothideomycetes; Capnodiales; Davidiellaceae
12th	Unclassfied_6	Ascomycota; Eurotiomycetes; Eurotiales; Unclassfied
13th	Unclassfied_18	Ascomycota; Sordariomycetes; Unclassfied; Unclassfied
14th	Incertae_sedis_13	Ascomycota; Dothideomycetes; Pleosporales; Incertae_sedis_13
15th	Helotiaceae	Ascomycota; Leotiomycetes; Helotiales; Helotiaceae
16th	Incertae_sedis_2	Ascomycota; Leotiomycetes; Helotiales; Incertae_sedis_2
17th	Herpotrichiellaceae	Ascomycota; Eurotiomycetes; Chaetothyriales; Herpotrichiellaceae
18th	Incertae_sedis_5	Basidiomycota; Incertae_sedis_4; Malasseziales; Incertae_sedis_5
19th	Incertae_sedis_1	Ascomycota; Leotiomycetes; Incertae_sedis; Incertae_sedis_1
20th	Incertae_sedis_12	Basidiomycota; Tremellomycetes; Tremellales; Incertae_sedis_12
	Others	Others

**Table A5:** The detailed taxonomic information of the top 20 fungi of soil samples on family rank.

Ranked List	Annotations in Figure 4h	Detailed Taxonomic Information
1st	Unclassfied_22	Basidiomycota; Unclassfied; Unclassfied; Unclassfied
2nd	Unclassfied_1	Unclassfied; Unclassfied; Unclassfied; Unclassfied
3rd	Helotiales_family_Incertae_sedis	Ascomycota; Leotiomycetes; Helotiales; Helotiales_family_Incertae_sedis
4th	Pyronemataceae	Ascomycota; Pezizomycetes; Pezizales; Pyronemataceae
5th	Hygrophoraceae	Basidiomycota; Agaricomycetes; Agaricales; Hygrophoraceae
6th	Leptosphaeriaceae	Ascomycota; Dothideomycetes; Pleosporales; Leptosphaeriaceae
7th	Unclassfied_2	Ascomycota; Unclassfied; Unclassfied; Unclassfied
8th	Pleosporales_family_Incertae_sedis	Ascomycota; Dothideomycetes; Pleosporales; Pleosporales_family_Incertae_sedis
9th	Ascomycota_family_Incertae_sedis	Ascomycota; Ascomycota_class_Incertae_sedis; Ascomycota_order_Incertae_sedis;family_Incertae_sedis
10th	Nectriaceae	Ascomycota; Sordariomycetes; Hypocreales; Nectriaceae
11th	Unclassfied_12	Ascomycota; Leotiomycetes; Helotiales;
12th	Mortierellaceae	Zygomycota; Zygomycota_class_Incertae_sedis; Mortierellales; Mortierellaceae
13th	Unclassfied_11	Ascomycota; Leotiomycetes; Unclassfied; Unclassfied
14th	Chaetomiaceae	Ascomycota; Sordariomycetes; Sordariales; Chaetomiaceae
15th	Davidiellaceae	Ascomycota; Dothideomycetes; Capnodiales; Davidiellaceae
16th	Venturiaceae	Ascomycota; Dothideomycetes; Venturiales; Venturiaceae
17th	Unclassfied_14	Ascomycota; Leotiomycetes; Thelebolales; Unclassfied
18th	Unclassfied_23	Basidiomycota; Agaricomycetes; Unclassfied; Unclassfied
19th	Myxotrichaceae	Ascomycota; Leotiomycetes; Leotiomycetes_order_Incertae_sedis; Myxotrichaceae
20th	Strophariaceae	Basidiomycota; Agaricomycetes; Agaricales; Strophariaceae
	Others	Others
